# Expression of Insulin-like growth factor 2 mRNA-binding protein 3 and its diagnostic value in breast cancer

**DOI:** 10.3389/fonc.2025.1624870

**Published:** 2025-07-02

**Authors:** Chao-Qun Wang, Jun-Kang Shao, Yan Wang, Shi-Ping Lu, Li-Jing Jiang, Zhi-Qun Du, Bi-Fei Huang

**Affiliations:** ^1^ Department of Pathology, Affiliated Dongyang Hospital of Wenzhou Medical University, Dongyang, Zhejiang, China; ^2^ Department of Medical Oncology, Affiliated Dongyang Hospital of Wenzhou Medical University, Dongyang, Zhejiang, China

**Keywords:** insulin-like growth factor 2 mRNA-binding protein 3, IGF2BP3, breast cancer, immunohistochemistry, prognosis

## Abstract

There is currently no established systematic explanation of the clinical application and diagnostic value of insulin-like growth factor 2 mRNA-binding protein 3 (IGF2BP3) in breast cancer. This study utilized immunohistochemistry (IHC) to assess the expression of the IGF2BP3 protein in 299 cases of breast cancer tissues, with the aim of investigating the clinical and diagnostic significance of IGF2BP3 in breast cancer. Our results showed that the positivity rate of IGF2BP3 in breast cancer tissues was 11.4% (34/299), significantly higher than that in normal breast tissues (0.0%, 0/60) (*P*=0.006). IGF2BP3 levels were found to be elevated in breast cancer cases with higher tumor grade, ER negative, PR negative, HER2 negative, higher Ki-67 index, and the triple-negative breast cancer (TNBC) molecular subtype (all *P*<0.05). Conversely, the presence of IGF2BP3 was less common in breast cancer cases with axillary lymph node metastasis and advanced tumor stage (both *P*<0.05). In a multiple logistic regression analysis, the independent predictors of IGF2BP3 expression in breast cancer were TNBC status (odds ratio = 3.408; 95% confidence interval: 1.026–11.321; *P*=0.045) and axillary lymph node metastasis (0.200; 0.068–0.593; *P*=0.004). We further analyzed the value of IGF2BP3 in the differential diagnosis of breast cancer and normal breast tissue, as well as the differential diagnosis of TNBC and non-TNBC. The results showed that the sensitivity, specificity, positive predictive value (PPV), and negative predictive value (NPV) of IGF2BP3 positive prediction for breast cancer were 11.4%, 100.0%, 100.0%, and 18.5%, respectively; and for TNBC, the corresponding values for IGF2BP3 positive prediction were 38.0%, 98.2%, 88.2%, and 81.5%, respectively. In all breast cancer or TNBC patients, no clear relationship between patient prognosis and IGF2BP3 expression was observed. We suggest that IGF2BP3 is upregulated in breast cancer, especially in TNBC, and has potential diagnostic value for breast cancer and TNBC.

## Introduction

Female breast cancer is the most frequently diagnosed cancer globally and is the top cause of cancer-related death in women (1, 2). There is considerable variation in prognosis and response to treatment. Triple-negative breast cancer (TNBC) is a specific subtype characterized by the absence of estrogen receptor (ER), progesterone receptor (PR), and human epidermal growth factor receptor 2 (HER2) expression. TNBC is linked to a poor prognosis because of its aggressive characteristics and limited targeted treatment options (3–6). Molecular subtyping of TNBC has been suggested as a potential approach for personalized treatment and predicting prognosis.

Insulin-like growth factor 2 mRNA-binding protein 3 (IGF2BP3) is a member of the IGF2 mRNA-binding protein family, which plays a crucial role in post-transcriptional regulation of gene expression (7, 8). Previous studies have suggested that IGF2BP3 is overexpressed in various malignant tumors and is associated with tumor progression, metastasis, and poor prognosis (8, 9). Currently, some studies have reported on the expression and clinical significance of IGF2BP3 in breast cancer. For example, in an IHC study of 138 invasive ductal carcinomas of the breast, tumors that were histologically high-grade, exhibited tumor necrosis, or were of the TNBC subtype showed significantly increased IGF2BP3 expression, and IGF2BP3 expression was associated with poor patient prognosis (10). In another study of 118 TNBC patients, positive IGF2BP3 expression was linked to larger tumor size, higher clinical stage, and basal morphology. Both disease-free survival and overall survival were significantly shorter in IGF2BP3-positive TNBC cases (11). A study comparing IGF2BP3 expression via IHC in 39 cases of invasive breast carcinoma with BRCA mutations and 54 cases of sporadic invasive breast carcinoma revealed that IGF2BP3 expression was more commonly observed in carcinomas with BRCA mutations (12). Furthermore, among 31 cases of metaplastic breast carcinoma, 13 cases showed high IGF2BP3 expression. The group with high IGF2BP3 expression was also associated with reduced overall survival compared to the low-expression group (13). Additionally, in the less common phyllodes tumors of the breast, IGF2BP3 expression was significantly increased in malignant phyllodes tumors compared to benign and borderline phyllodes tumors (14, 15).

However, a systematic explanation of the clinical application and diagnostic value of IGF2BP3 in breast cancer has not been achieved, mainly due to limited sample sizes in some studies or insufficient comprehensive clinicopathological correlation analyses. In this study, we aim to investigate the expression of IGF2BP3 in breast cancer tissues and its clinical significance. We will use immunohistochemistry (IHC) to analyze the IGF2BP3 protein expression in 299 cases of breast cancer. The results of this study may provide valuable insights into the role of IGF2BP3 in breast cancer progression and its potential as a diagnostic and prognostic marker. Understanding the role of IGF2BP3 may lead to the development of novel targeted therapies for breast cancer patients, particularly those with TNBC who currently have limited treatment options.

## Materials and methods

### Patients and tissue samples

Tissue samples of breast cancer were collected from 299 Chinese Han women who had undergone surgery for breast cancer at the Affiliated Dongyang Hospital of Wenzhou Medical University (Dongyang, Zhejiang, China) from 2007 to 2018. Contains 289 cases of invasive ductal carcinoma, 5 cases of medullary carcinoma and 5 cases of metaplastic carcinoma. Sixty samples of adjacent normal breast tissue were also obtained following surgical resection. Patients included in the study were those admitted to the hospital and underwent surgery, with pathological confirmation of invasive breast cancer, and the participants or the participants’ legal guardians/next of kin agreed to participate in the scientific research. Patients who had received anti-tumor treatments prior to surgery, including targeted therapy, chemotherapy, radiotherapy, or immunotherapy, were excluded from the study. Breast cancer patients were aged between 24 and 84 years, with a median age of 51 years. A pathohistological diagnosis was made according to breast tumor classification criteria of the World Health Organization (16, 17). Histological grading was based on the Scarff-Bloom-Richardson system (18). According to ER, PR and HER2 status, tissue samples were classified into triple-negative breast cancer (TNBC) (ER^–^, PR^–^, HER2^–^) or non-TNBC subtype (19–22). The characteristics and grouping information of breast cancer patients can be found in **Table 1**. Among them, information on the ki-67 index was missing for 5 cases. Follow-up information was available for 273 patients with a median follow-up time of 60 months (range, 4–60 months). All of the study methodology satisfied the relevant guidelines and regulations issued by the Affiliated Dongyang Hospital of Wenzhou Medical University.

**Table 1:** Association of IGF2BP3 expression with clinicopathological parameters in breast cancer patients.

Variables	No. of patients	IGF2BP3 positive, n (%)	*P*-value*
Age (years)			
≤35	14	3 (21.4%)	0.426
36–55	177	18 (10.2%)	
>55	108	13 (12.0%)	
Tumor size (cm)			
≤2	143	14 (9.8%)	0. 410
>2	156	20 (12.8%)	
Lymph node metastases			
No	156	29 (18.6%)	**<0.001**
Yes	143	5 (3.5%)	
Tumor grade			
I	13	0 (0.0%)	**<0.001**
II	183	9 (4.9%)	
III	103	25 (24.3%)	
Tumor stage			
I	86	13 (15.1%)	**0.023**
II	151	20 (13.2%)	
III	62	1 (1.6%)	
Estrogen receptor			
Negative	131	34 (26.0%)	**<0.001**
Positive	168	0 (0.0%)	
Progesterone receptor			
Negative	162	33 (20.4%)	**<0.001**
Positive	137	1 (0.7%)	
HER2 expression			
Negative (0–1^+^)	138	27 (19.6%)	**<0.001**
Equivocal (2^+^)	91	5 (5.5%)	
Positive (3^+^)	70	2 (2.9%)	
Ki-67			
<14%	125	0 (0.0%)	**<0.001**
≥14%	169	34 (20.1%)	
Molecular classification			
non-TNBC	220	4 (1.8%)	**<0.001**
TNBC	79	30 (38.0%)	
Lymphvascular invasion			
No	129	19 (14.7%)	0.059
Yes	29	0 (0.0%)	

*Pearson’s chi-square test is used for the comparison of the IGF2BP3 positive rate among different groups. A bold value of *P*<0.05 indicates statistical significance.

### Tissue array preparation and IHC analysis

Tissue Array Preparation: We followed the methods described by Wang et al., 2020 (23). To summarize, the Quick-Ray^®^ UT-06 tissue microarray system and the Quick-Ray premade recipient block (UB-06) wax model, both produced by Unitma Co., Ltd. in Seoul, Korea, were utilized for the preparation of tissue specimens measuring 1 mm in diameter. Two specific locations were chosen from each sample of breast cancer tissue for sampling purposes. IHC Analysis: The Envision System (Dako, Glostrup, Denmark) was used for IHC staining of paraffin-embedded tissue sections, following the method previously described (20, 21). The primary antibodies utilized in the experiment were the anti-IGF2BP3 rabbit monoclonal antibody (clone EPR12021-114) obtained from Abcam in Cambridge, England, diluted to a concentration of 1:400. Also used were the ready-to-use anti-ER rabbit monoclonal antibody (clone SP1) from Dako in Glostrup, Denmark, the ready-to-use anti-PR mouse monoclonal antibody (clone PgR636) from Dako, the ready-to-use anti-Ki-67 mouse monoclonal antibody (clone MIB-1) from Dako, the ready-to-use anti-Podoplanin mouse monoclonal antibody (clone D2-40) from Dako, and HercepTest from Dako. For the secondary antibody, Dako’s HRP rabbit/mouse universal antibody (from Dako, Glostrup, Denmark) was employed. The vehicle was incubated first in the negative control, then the secondary antibody was added without any primary antibody. IGF2BP3-positive breast cancer tissue was utilized as the positive control.

### Assessment of staining

IGF2BP3 is predominantly found in the cytoplasm of breast cancer cells. In this research, any staining present in ≥1% of breast cancer cells or normal glandular epithelial cells is categorized as IGF2BP3 positive (24). A case was considered to be ER- or PR-positive when the percentage of positive invasive cancer cells (nuclear staining) was ≥1% (25). HER2 status was determined by the 2018 American Society of Clinical Oncology/College of American Pathologists guidelines for HER2 testing in breast cancer (26). The interpretation of staining results is independently completed by two pathologists.

### Patient follow-up

Patients were followed-up using previously described methods (22, 23). In conclusion, every patient was monitored post-operation via phone calls and at hospital visits every 6 months; follow-up would cease upon the patient’s passing or was lost to follow-up. Breast cancer recurrence was identified using clinical imaging or pathological histology. Relapse-free survival (RFS) was calculated as the time from surgery to relapse or metastasis, distant metastasis-free survival (DMFS) was measured from surgery to metastasis, and overall survival (OS) was defined as the time from surgery to death (excluding deaths unrelated to the tumor).

### Statistical analysis

Statistical analysis was carried out using SPSS software version 19.0 (SPSS Inc, Chicago, IL, USA). Differences in IGF2BP3 expression among groups were compared using a Pearson’s chi-square test for categorical variables. Multivariate logistic regression analysis was utilized to identify independent correlation factors of IGF2BP3 expression. The predictive ability of IGF2BP3 for breast cancer or TNBC was assessed by calculating its sensitivity, specificity, positive predictive value (PPV), and negative predictive value (NPV). RFS, DMFS and OS rates were determined using the Kaplan-Meier method and compared with log-rank testing. Multivariate analysis using the Cox proportional hazard model was performed to investigate independent factors prognostic of RFS, DMFS and OS. A significance level of *P*<0.05 was used for statistical analysis.

## Results

### IGF2BP3 expression in breast tissues

**Figure 1:**
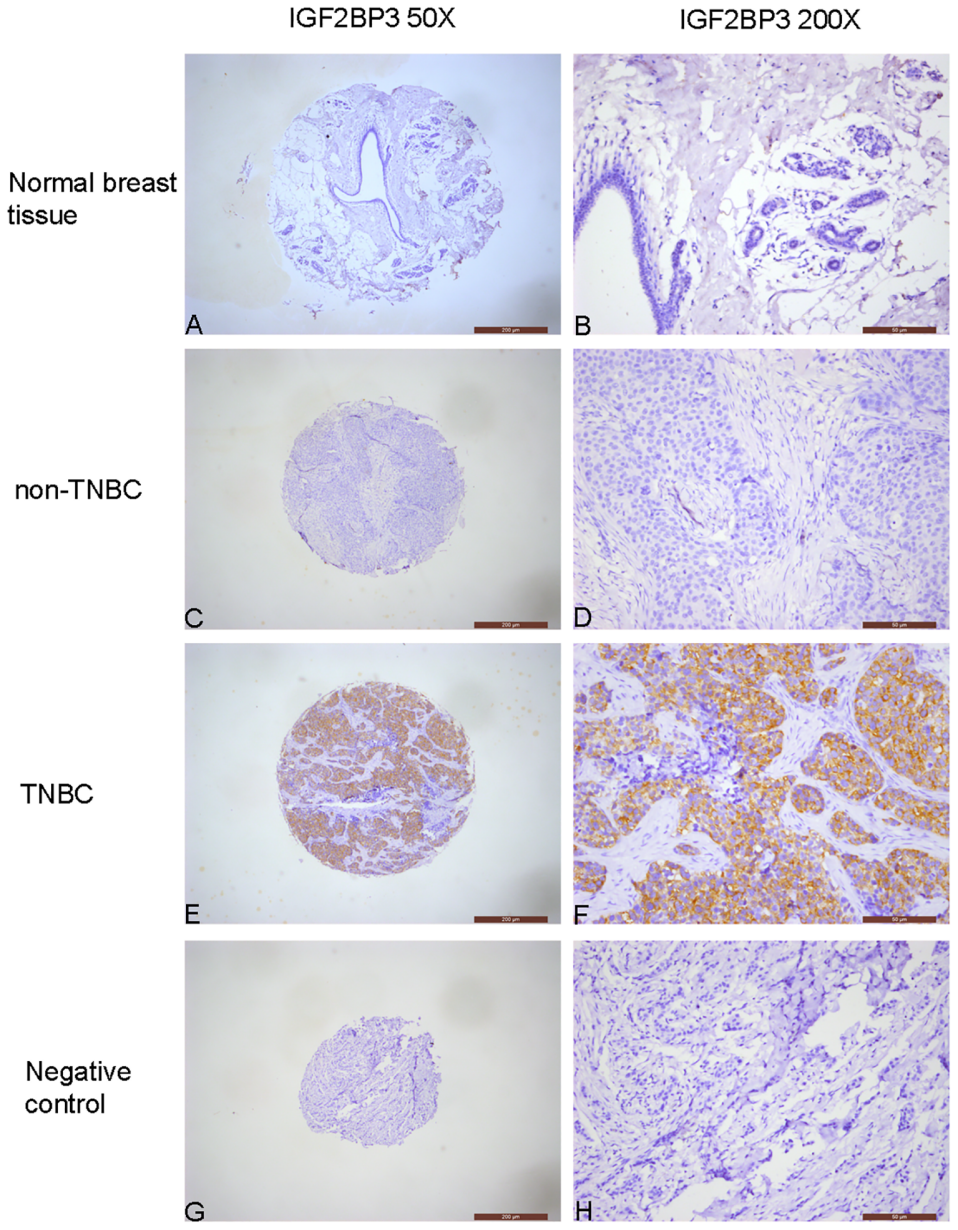
Immunochemical analysis of IGF2BP3 expression in breast tissues. **(A, B)** Normal breast tissue, negative IGF2BP3 expression in normal glandular epithelial cells. **(C, D)** non-TNBC, negative IGF2BP3 expression in cancer cells. **(E, F)** TNBC, positive IGF2BP3 expression in cancer cells. **(G, H)** Negative control, all cells in the breast cancer tissue, including cancer cells and stromal cells, show negative expression of IGF2BP3.

Among 299 cases of breast cancer tissues, 34 were IGF2BP3 positive (11.4%, 34/299), while the IGF2BP3 positivity rate in 60 cases of normal breast tissues was 0.0% (0/60) (**Table 2**). The IGF2BP3 positivity rate in breast cancer tissues was significantly higher than in normal breast tissues (*P*=0.006) (**Figure 1**).

**Table 2:** IGF2BP3 expression in breast tissue specimens.

Tissue samples	No.	IGF2BP3 expression	
		Negative, n (%)	Positive, n (%)
Noncancerous	60	60 (100.0%)	0 (0.0%)
Cancerous	299	265 (88.6%)	34 (11.4%)*

*P<0.05 vs normal breast tissue.

### Relationship between IGF2BP3 expression and clinicopathological features of breast cancer

**Table 1** shows that the positivity rate of IGF2BP3 in grade III breast cancer patients (24.3%, 25/103) was significantly higher compared to patients in grades II (4.9%, 9/183) and I (0.0%, 0/13) (*P*<0.001). Likewise, the positivity rates of IGF2BP3 in ER negative, PR negative, and breast cancer patients with a higher Ki-67 index (≥14%) were 26.0% (34/131), 20.4% (33/162), and 20.1% (34/169) respectively, all significantly higher than those in ER positive (0.0%, 0/168), PR positive (0.7%, 1/137), and low Ki-67 index (<14%) (0.0%, 0/125) patients (all *P*<0.001). Furthermore, the positivity rate of IGF2BP3 in HER2 negative (0-1+) breast cancer patients (19.6%, 27/138) was significantly higher compared to patients with HER2 equivocal (2+) (5.5%, 5/91) and HER2 positive (3+) (2.9%, 2/70) (*P*<0.001). Lastly, among 79 TNBC cases, 30 (38.0%) showed IGF2BP3 positivity, significantly higher than the 1.8% (4/220) observed in non-TNBC (*P*<0.001).

Interestingly, we found that the positivity rate of IGF2BP3 in breast cancer without axillary lymph node metastasis (18.6%, 29/156) was significantly higher than that in patients with axillary lymph node metastasis (3.5%, 5/143) (*P*<0.001). Furthermore, the positivity rates of IGF2BP3 in stage I, II, and III breast cancer were 15.1% (13/86), 13.2% (20/151), and 1.6% (1/62) respectively, with a statistically significant difference between groups (*P*=0.023). To further verify the relationship between IGF2BP3 expression and lymph node metastasis, we examined the postoperative pathological results of breast cancer cases. Among them, 158 cases underwent D2–40 IHC detection, with 29 cases showing lymphatic vessel invasion (LVI). We then proceeded to analyze the correlation between IGF2BP3 expression and lymphatic invasion. Similar to the axillary lymph nodes findings, the rate of IGF2BP3 positivity in patients without LVI was 14.7% (19/129), significantly higher than the 0.0% (0/29) in patients with LVI, although no statistical significance was observed (*P*=0.059).

The results of the multiple logistic regression analysis showed that TNBC status (odds ratio [*OR*] = 3.408; 95% confidence interval [*CI*]: 1.026–11.321; *P*=0.045) and axillary lymph node metastasis (0.200; 0.068–0.593; *P*=0.004) were the independent predictors of IGF2BP3 expression in breast cancer.

### The value of IGF2BP3 expression for diagnosing breast cancer or TNBC

**Figure 2:**
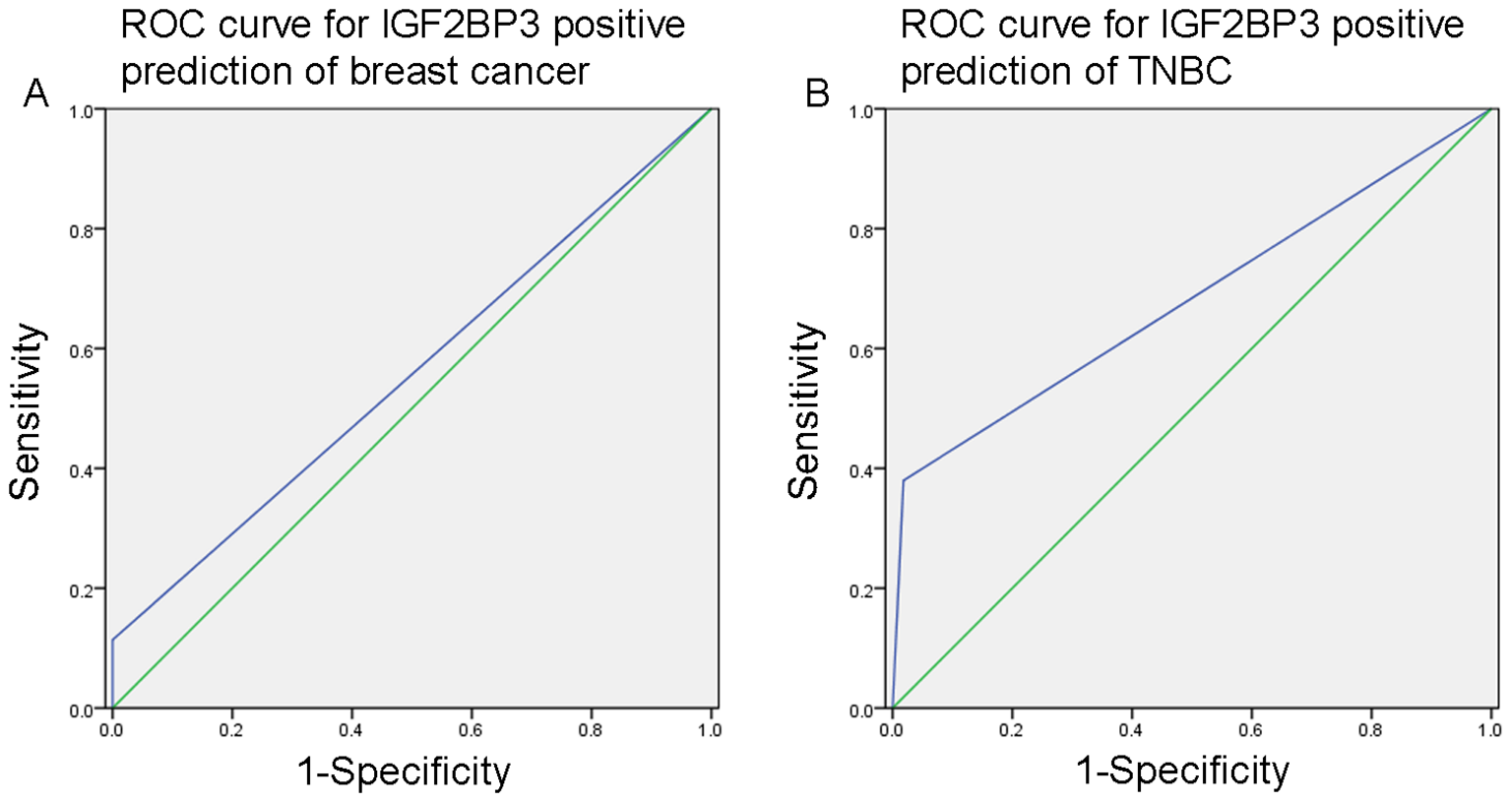
**(A)** The ROC curve analysis indicated that the area under the curve (AUC) for predicting breast cancer with positive IGF2BP3 expression was 0.557 (95% *CI* 0.483–0.631; *P*=0.164). **(B)** The AUC for predicting TNBC with positive IGF2BP3 expression was 0.681 (95% *CI* 0.604–0.758; *P*<0.001).

We further analyzed the value of IGF2BP3 in the differential diagnosis of breast cancer and normal breast tissue, as well as the differential diagnosis of TNBC and non-TNBC. The ROC curve analysis showed that the area under the curve (AUC) for IGF2BP3 positive prediction of breast cancer was 0.557 (95% *CI* 0.483–0.631; *P*=0.164; **Figure 2A**), with a sensitivity of 11.4%, specificity of 100.0%, PPV of 100.0%, and NPV of 18.5%. Additionally, the AUC for IGF2BP3 positive prediction of TNBC was 0.681 (0.604–0.758; *P*<0.001; **Figure 2B**), with a sensitivity of 38.0%, specificity of 98.2%, PPV of 88.2%, and NPV of 81.5%.

### No association between IGF2BP3 expression and survival

**Figure 3:**
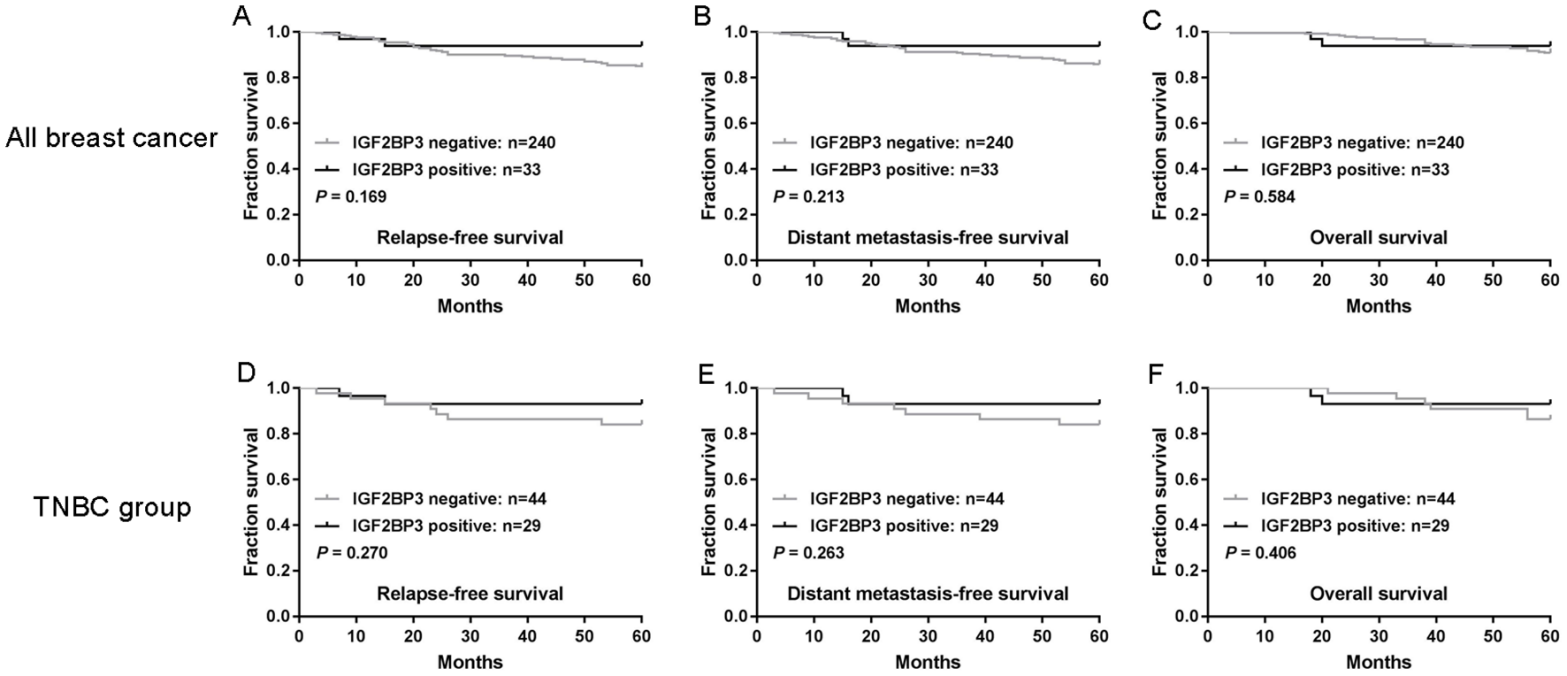
IGF2BP3 expression is not associated with the survival of patients with breast cancer. **(A-C)** The associations of IGF2BP3 expression with relapse-free survival (RFS) **(A)**, distant metastasis-free survival (DMFS) **(B)** and overall survival (OS) **(C)**. **(D-F)** The associations of IGF2BP3 expression with RFS **(D)**, DMFS **(E)** and OS **(F)** of patients with TNBC. *P*-values were calculated using the Mantel-Cox log-rank test.

To assess the potential impact of IGF2BP3 expression on patient survival, we analyzed IGF2BP3 expression in relation to RFS, DMFS and OS rates in patients with breast cancer. Five-year RFS, DMFS and OS rates were 85.7%, 86.8% and 91.2%, respectively. As shown in **Figures 3A–C**, no clear associations were observed between IGF2BP3 expression and these survival variables (*P*>0.05 for each comparison).

We further analyzed the effect of IGF2BP3 expression on the prognosis of TNBC. As shown in **Figures 3D–F**, in TNBC, the prognosis of tumors that were positive IGF2BP3 expression did not differ significantly from that of negative IGF2BP3 group (*P*>0.05 for each comparison).

In Cox proportional hazards regression analysis, the results showed that compared to individuals ≤35 years old, patient age between 36–55 years old is an independent protective factor for RFS (hazard ratio [*HR*]=0.266, 95% *CI*=0.074–0.955, *P*=0.042), DMFS (0.239, 0.066–0.869, *P*=0.030) and OS (0.090, 0.021–0.390, *P*=0.001); On the other hand, stage III tumor is an independent unfavorable prognostic factor for RFS (5.710, 2.074–15.722, *P*=0.001), DMFS (5.297, 1.905–14.726, *P*=0.001) and OS (22.812, 2.913–178.623, *P*=0.003).

## Discussion

IGF2BP3 plays an important role in regulating gene expression after transcription (7, 8). Previous research has indicated that IGF2BP3 is often overexpressed in different types of cancer, and is linked to the advancement of tumors, their spread to other parts of the body, and poor prognosis (8, 9). While some studies have looked into the expression and significance of IGF2BP3 in breast cancer (10–15), a comprehensive understanding of its clinical use and diagnostic value in this type of cancer has not been reached, possibly due to limited sample sizes in some studies or insufficient comprehensive clinicopathological correlation analyses.

The results of our study suggest that IGF2BP3 expression is associated with specific clinicopathological characteristics of breast cancer, and the positivity rate of IGF2BP3 is significantly higher in breast cancer tissues compared to normal breast tissues highlights the potential role of IGF2BP3 in the development of breast cancer. Our results show that high levels of IGF2BP3 are linked to higher tumor grade, ER negativity, PR negativity, HER2 negativity, higher Ki-67 index, and the TNBC molecular subtype. These results are relatively consistent and easy to understand, because TNBC is a subtype of breast cancer that is negative for ER/PR/HER2, and is associated with high tumor grade and Ki-67 index (3–6). Due to these characteristics, TNBC is usually considered to have aggressive characteristics, poor prognosis, and limited targeted treatment options (3–6). These findings suggest that IGF2BP3 may contribute to the aggressiveness of breast cancer and could be a potential biomarker for identifying more aggressive subtypes of the disease.

We found a significant correlation between IGF2BP3 expression and the presence of axillary lymph node metastasis, with lower expression in patients with metastasis. Furthermore, our analysis of D2–40 detection data showed that IGF2BP3 positivity was also higher in patients negative for lymphatic vessel invasion compared to those positive for lymphatic vessel invasion. The multiple logistic regression analysis further confirms TNBC status and axillary lymph node metastasis as independent predictors of IGF2BP3 expression. Studies show that TNBC subtype had lower odds of LVI (27, 28) and axillary lymph node involvement (28–31) relative to other subtypes. Therefore, it may be one of the explanations for the low incidence of lymph node metastasis in breast cancer with positive IGF2BP3 expression.

This study focused on exploring the diagnostic value of IGF2BP3 in breast cancer, particularly in TNBC. The results showed that the performance of IGF2BP3 in the differential diagnosis of breast cancer was limited, with an AUC of 0.557. Although it had high specificity (100.0%), its sensitivity was low (11.4%), which restricts its potential as a comprehensive diagnostic tool. However, in TNBC, IGF2BP3 showed better diagnostic value, with an AUC of 0.681, good specificity (98.2%), and PPV (88.2%). Notably, the limited number of analyzed TNBC patients limits the strength of this ROC analysis. These characteristics highlight the potential application value of IGF2BP3 as a supplementary biomarker in the diagnosis of TNBC.

Although IGF2BP3 showed some value in the diagnosis of TNBC, its role in prognosis was not significant. There was no significant correlation between the expression level of IGF2BP3 and RFS, DMFS, or OS in both all breast cancer patients and TNBC patients. This indicates that IGF2BP3 may not be a prognostic indicator. Whether extending the follow-up duration or increasing the sample size of TNBC will have a meaningful positive impact on the prognostic value of IGF2BP3 remains to be further confirmed by future studies.

In conclusion, these findings suggest that IGF2BP3 has the potential to be a biomarker for identifying more aggressive subtypes of breast cancer, particularly TNBC, and it has good clinical value in diagnosing TNBC. However, there are limitations in the study of IGF2BP3 in breast cancer, including a retrospective design that may introduce biases, lack of functional studies on IGF2BP3’s role in cancer progression, limited evidence for its prognostic value, and the relatively small number of positive cases. Future multi-center studies with expanded cohorts, combined with functional experiments and longitudinal survival analysis, should further explore the biological role and clinical potential of IGF2BP3 to provide more practical guidance for the diagnosis and treatment of TNBC.

## Data Availability

The original contributions presented in the study are included in the article/Supplementary Material, further inquiries can be directed to the corresponding author/s.
